# Astragaloside IV Alleviates Cerebral Ischemia-Reperfusion Injury through NLRP3 Inflammasome-Mediated Pyroptosis Inhibition via Activating Nrf2

**DOI:** 10.1155/2021/9925561

**Published:** 2021-12-30

**Authors:** Lan Xiao, Ziwei Dai, Wenjing Tang, Canwen Liu, Biao Tang

**Affiliations:** Hunan Provincial Key Laboratory of Pathogenic Biology Based on Integrated Chinese and Western Medicine, Hunan University of Chinese Medicine, Changsha 410208, China

## Abstract

As one of the fundamental components of *Astragalus membranaceus*, astragaloside IV (AST IV) exerts protective effects against cerebral ischemia-reperfusion injury (CIRI). Nevertheless, the underlying mechanisms have not yet been conclusively elucidated. To do so, here, we report on the regulatory effects of Nrf2 on NLRP3 inflammasome-mediated pyroptosis. CIRI was induced by middle cerebral artery occlusion-reperfusion (MCAO/R) in Sprague Dawley rats and modeled by oxygen and glucose deprivation/reoxygenation (OGD/R) in SH-SY5Y cells. Cerebral infarct volume and neurological deficit score served as indices to evaluate MCAO/R injury. In addition, the CCK-8 assay was used to assess cell viability, the LDH leakage rate was used as a quantitative index, and propidium iodide (PI) staining was used to visualize cells after OGD/R injury. The NLRP3/Caspase-1/GSDMD pathway, which produces the pores in the cell membrane that are central to the pyroptosis process, was assessed to investigate pyroptosis. Nrf2 activation was assessed by detecting Nrf2 protein levels and immunofluorescence analysis. We show that after MCAO/R of rats, the infarct volume and neurological deficit score of rats were strongly increased, and after OGD/R of cell cultures, cell viability was strongly decreased, and the LDH leakage rate and the proportion of PI-positive cells were strongly increased. In turn, MCAO/R and OGD/R enhanced the protein levels of NLRP3, Caspase-1, IL-1*β*, GSDMD, and GSDMD-N. Moreover, Nrf2 protein levels increased, and Nrf2 translocation was promoted after CIRI. Interestingly, AST IV (i) reduced the cerebral infarct volume and the neurological deficit score in vivo and (ii) increased the cell viability and reduced the LDH leakage rate and the proportion of PI-positive cells in vitro. AST IV reduced the protein levels of NLRP3, Caspase-1, IL-1*β*, GSDMD, and GSDMD-N, inhibiting NLRP3 inflammasome-mediated pyroptosis. AST IV also increased the protein levels of Nrf2 and promoted the transfer of Nrf2 to the nucleus, accelerating Nrf2 activation. Particularly revealing was that the Nrf2 inhibitor ML385 partly blocked the above effects of AST IV. Taken together, these results demonstrate that AST IV alleviates CIRI through inhibiting NLRP3 inflammasome-mediated pyroptosis via activating Nrf2.

## 1. Introduction

Stroke is the number one disease reducing people's quality of life worldwide, with ischemic stroke being the most frequent cause of death and disability. Currently, thrombolysis and thrombectomy are the preferred treatment methods for ischemic stroke. Noteworthily, the recovery of blood flow (reperfusion) aggravates the injury, resulting in cerebral ischemia-reperfusion injury (CIRI). CIRI affects the prognosis of ischemic stroke, and it is the main focus in the prevention and treatment of ischemic stroke [1].

The pathological process underlying CIRI is composed of numerous links, mainly involving the inflammatory response, free radical injury, excitatory amino acid toxicity, mitochondrial energy metabolism disorder, and pyroptosis. All links interact with each other, interweaving into an intricate and huge regulatory network, triggering a series of pathological cascade reactions, which directly or indirectly lead to neuronal death [2, 3].

Inflammation is one of the pathological processes essential to CIRI. It has previously been observed that pyroptosis mediated by the nucleotide-binding oligomerization domain-like receptor protein 3 (NLRP3) inflammasome is a prominent link in the inflammatory response of CIRI, with the NLRP3/Caspase-1/GSDMD pathway playing an important role [4, 5]. The NLRP3 inflammasome consists of NLRP3, apoptosis-associated speck-like protein containing CARD (ASC), and procysteinyl aspartate-specific protease-1 (pro-Caspase-1). After NLRP3 is recognized, its protein conformation changes. Then, it is recruited and combines with ASC through the homotypic interaction of its own domain, and then pro-Caspase-1 is recruited and cleaved by ASC to assemble the NLRP3 inflammasome. On the one hand, activated Caspase-1 is able to cleave activated proinflammatory cytokines interleukin-18 (IL-18) and IL-1*β*. On the other hand, it is capable of cleaving Gasdermin D (GSDMD). The N-terminal fragment of its product is lipophilic and binds to the cell membrane to form pores, leading to cell membrane perforation, activating pyroptosis. The release of proinflammatory factors aggravates the inflammatory response [6–9]. A number of studies have investigated whether the NLRP3/Caspase-1/GSDMD pathway mediates CIRI and whether pathway inhibition suppresses pyroptosis, alleviating injury [10, 11]. Therefore, NLRP3 inflammasome-mediated pyroptosis is likely to be involved in the mechanisms underlying CIRI.

Nrf2 is a substantial antioxidant transcription factor acting in the antioxidative stress system, which is able to reduce CIRI by exerting anti-inflammatory effects, inhibiting oxidative stress and neuronal apoptosis, and promoting angiogenesis [12–14]. Moreover, Nrf2 has been shown to negatively regulate NLRP3 inflammasome activation and hence plays a significant role in CIRI. Thus far, several lines of study have shown that Nrf2 prevents NLRP3 inflammasome activation by regulating reactive oxygen species (ROS) levels, the thioredoxin system, and the glutathione protein system, decreasing the oxidative stress level [14, 15]. Some studies have also revealed that Nrf2 activation inhibits the NLRP3/Caspase-1/GSDMD pathway and pyroptosis in renal ischemia-reperfusion injury [16–18]. Consequently, negative regulatory effects of Nrf2 on NLRP3 inflammasome-mediated pyroptosis may be a valuable way to prevent or treat CIRI.

Astragaloside IV (AST IV) is an effective component and quality indicator of *Astragalus membranaceus*. It is widely used in the prevention and treatment of cardiovascular and cerebrovascular diseases in China [19, 20]. Many studies have demonstrated it reduces CIRI by exerting anti-inflammatory, antiapoptotic, and antioxidative effects [21–28]. It has been verified that it reduces CIRI by activating Nrf2 and inhibiting the inflammatory response, among others [29–32]; yet, its specific mechanism of action remains to be elucidated.

Accordingly, in the present study, an in vivo CIRI rat model was established by middle cerebral artery occlusion/reperfusion (MCAO/R), and SH-SY5Y cells were treated with oxygen and glucose deprivation/reoxygenation (OGD/R) to construct an in vitro model. Based on the regulatory effects of Nrf2 on pyroptosis mediated by the NLRP3 inflammasome, the mechanisms underlying the effects of AST IV on CIRI were further explored to gain a better understanding of (i) how the NLRP3/Caspase-1/GSDMD pathway is activated upon CIRI to promote pyroptosis and aggravate the injury and (ii) how AST IV inhibits NLRP3-mediated pyroptosis by activating Nrf2 to alleviate CIRI.

## 2. Materials and Methods

### 2.1. Animal Grouping and Model Establishment

We purchased healthy adult male Sprague Dawley rats weighing 200–220 g (specific pathogen-free [SPF] grade) from Hunan Slake Jingda Experimental Animal Co., Ltd. (Animal Certificate No. SCXK, Xiang, 2019-0004). Animals were housed under SPF conditions at the Experimental Animal Center of Hunan University of Chinese Medicine (HUCM) on a 12/12-h light/dark cycle, at an ambient temperature of 25 ± 1°C, relative humidity of 60%, and a wind speed of 0.3 m/s. After 1 week of adaptive feeding (12.5 g rat food every 12 h), rats were fasted with free access to water for 12 h prior to the experiments. All of the used protocols, such as study design, sample size, randomization, outcome measures, data analysis, experimental procedures, and reporting of results, were approved by the Animal Ethics Committee of HUCM (Changsha, China; Approval No. LL2021042803), were in compliance with the Guide for the Care and Use of Laboratory Animals published by the US National Institutes of Health (NIH publication No. 85–23, revised 1996), and were in agreement with the ARRIVE guidelines. All of the methods were performed in accordance with the relevant guidelines and regulations.

Rats were randomly divided into four groups: (i) the sham group, (ii) the MCAO/R group, (iii) the AST IV group, and (iv) the AST IV + ML385 group. Rats in the AST IV and AST IV + ML385 groups received AST IV three times (10 ml/kg per time) by gavage. Optimal administration times were 50 h, 26 h, and 2 h prior to model establishment. The sham and MCAO/R groups received equal volumes of physiological saline. The MCAO/R model was established as previously described. Briefly, after intraperitoneal injection of 2% sodium pentobarbital, the anterior median skin of rats was cut, and the left common carotid artery (CCA), external carotid artery (ECA), and internal carotid artery (ICA) were separated. The distal end of the ECA was ligated by a surgical line, and the ECA and its branches were coagulated by an electrocoagulation pen near the ligation point. The distal ICA and CCA were clipped by the artery. A small orifice was cut at the stump of the ECA, a suture was inserted from the ECA through the CCA bifurcation into the ICA, the artery clamp on the ICA was loosened, and the suture into the intracranial ICA segment was inserted; the length of the line was about (18 ± 2) mm from the CCA bifurcation. The suture in the ICA was ligated to prevent bleeding and movement of the suture, and wounds were sutured. After blocking the blood flow for 2 h, the suture was pulled out for reperfusion for 24 h [26, 33]. In the sham group, only the CCA, ECA, and ICA were dissociated, without thread insertion. Animal experiments were conducted in accordance with the “Guiding opinions on treating experimental animals kindly” issued by the Ministry of Science and Technology and approved by the Ethics Committee of Hunan University of Traditional Chinese Medicine.

A total of 86 rats were used, 5 of which died due to unplanned hemorrhage during surgery; 6 died due to intracranial hemorrhage and brain injury after surgery, and the remaining rats survived until euthanasia. Ten rats that underwent MCAO/R but did not show infarction due to surgical errors were also excluded from the study. Each group included 15 rats: 10 for 2,3,5-triphenyl tetrazolium chloride (TTC) staining and 5 for western blot analysis. Following this, the rats were euthanized by i.p. injection of excessive sodium pentobarbital (140 mg/kg body weight). Death was monitored based on cardiac activity and respiration. All efforts were taken to minimize animal suffering and reduce the number of animals used.

### 2.2. Neurologic Deficit Scoring

After 24 h of reperfusion, according to the Longa method [26, 34], the neurological deficit score was evaluated by experienced testers. To reduce bias, a double-blind design was used (the experimental personnel did not know which group was analyzed). Neurological deficit scores were recorded as follows: 0 = no neurological deficit symptoms, normal activity; 1 = the contralateral forelimb of the lesion could not be fully straightened; 2 = rats turned to the opposite side when crawling; 3 = rats walked their body to the opposite side; and 4 = rats were unable to walk on their own, loss of consciousness.

### 2.3. Cerebral Infarct Volume Measurement

The cerebral infarct volume was measured according to a previous study[26, 33]. The whole-brain tissues were harvested and frozen at −20°C for 10 min. Brain slices were placed in 2% TTC (Sigma, BCBP3272V) in phosphate buffer at 37°C. After staining, photographs were taken and the volume of cerebral infarcts was calculated.

### 2.4. Cell Culture and Establishment of the OGD/R Model

SH-SY5Y neuroblastoma cells were purchased from the Shanghai Institute of Cell Biology. Cells were cultured in high glucose RPMI-1640 medium containing 10% fetal bovine serum (Gibco, batch number: C11875500BT) and incubated at 37°C in 5% CO_2_. The following cell culture groups were established: the sham group, the OGD/R group, high-dose, medium-dose, and low-dose AST IV groups, the AC-YVAD-CMK (Sigma-Aldrich, USA, Batch No.: SML0429) group, the MCC950 (Selleck, USA, Batch No.: S7809) group, and the AST IV + ML385 group. After the cells were washed with DPBS, the OGD/R group was treated with 37°C preheated glucose-free DMEM, and air (95% N_2_ and 5% CO_2_) was continuously ventilated for 1.5 L/min for 6 min. After ventilation, the hypoxic culture chamber was closed. Cells were placed in the incubator for 2 h. After the treatment, the glucose-free DMEM (Gibco, USA, Batch No.: 21922567) was removed, and a complete medium was added. Then, cells were put into the incubator for 24 h for reoxygenation. The sham group was cultured in a complete culture medium. Cells in the AST IV intervention groups were cultured in a medium with different concentrations of AST IV (5, 10, 20, 40, or 80 *μ*g/ml), cells in the AC-YVAD-CMK group were treated with AC-YVAD-CMK, and cells in the MCC950 group were treated with MCC950.

### 2.5. CCK-8 Assay and LDH Release Assay

The cells were inoculated in a 96-well plate and allowed to adhere before model establishment. After drug administration, the CCK-8 kit (Dalian Meilun Biotechnology Co., Ltd., Batch No.: MA0218-1) was used to detect cell viability. The reagents diluted to working solution concentrations were added to the cells, which were incubated in the dark for 1 h. The absorbance at 450 nm was measured by an enzyme-labeled instrument. Relative cell viability was calculated as follows: relative cell viability (%) = (average absorbance of sample − average absorbance of the blank group)/(average absorbance of the normal group − average absorbance of the blank group) × 100%. Four parallel wells were included for each group of cells, and the experiment was performed in triplicate. The supernatant of each group was added to a new 96-well plate (60 *μ*L per well), and then 60 *μ*L LDH detection fluid was added to each well (Shanghai Biyuntian Biotechnology Co., Ltd., batch number: 060319190906). After incubation at room temperature for 30 min, the absorbance at 490 nm was detected by an enzyme-labeled instrument. LDH leakage rate was calculated as follows: LDH leakage rate (%) = (average sample absorbance − average blank group absorbance)/(average cell maximum enzyme activity well absorbance − average blank group absorbance) × 100%. The experiment was repeated three times.

### 2.6. PI and Hoechst 33342 Staining

SH-SY5Y cells were inoculated on a 24-well plate and allowed to adhere before model establishment. After the cells were treated, 5 *μ*L Hoechst 33342 staining solution and 5 *μ*L propidium iodide (PI) staining solution were added to each well (Shanghai Beyotime Biotechnology Co., Ltd., Batch No.: 101819200624). Cells were incubated at 4°C for 20 min and then observed under a fluorescence microscope. Three nonoverlapping fields were randomly taken and photographed. The proportion of PI-positive cells was calculated as follows: proportion of PI − positive cells (%) = (number of red fluorescent cells/total cells) × 100%. Three wells were set in each group, and the experiment was repeated three times.

### 2.7. Reactive Oxygen Species Assay

According to the instructions of the ROS detection kit, cells were inoculated in 24-well plates. After model establishment and drug administration, 200 *μ*L/well DCFH-DA (1 : 1000) diluted in serum-free culture medium was added (Shanghai Beyotime Biotechnology Co., Ltd., Batch No.: 041720200803), and cells were incubated at 37°C for 20 min. The cells were washed three times with a serum-free culture medium and photographed under a fluorescence microscope. Three wells were included per group, and three nonoverlapping fields of view were randomly selected and photographed. Quantitative analysis of ROS levels was conducted using ImageJ software.

### 2.8. Immunofluorescence

Following model establishment and drug administration, cells were fixed on cover slides with 4% paraformaldehyde (Beyotime, Batch No.: P0099) for 15 min, and the slides were washed with PBS three times, treated with 0.5% Triton X-100 (Beyotime, batch number: P0096) in PBS at room temperature for 20 min, washed with PBS three times, and blocked with BSA at 37°C for 1 h. The blocking solution was removed by absorbent paper, and a sufficient amount of primary antibody (1 : 100) was dropped on the slide (Affinity Biosciences, Batch No. AF0639). Samples were incubated overnight at 4°C and washed with PBST three times. Avoiding light, secondary antibody (1 : 200) (P0186) was added, and cells were incubated at 37°C for 1 h. Next, samples were washed with PBST three times, and cells were stained with DAPI (P0131, batch number) in the dark for 10 min and photographed under a fluorescence microscope. Each group included three wells, and three nonoverlapping fields of view were randomly selected and photographed. The fluorescence images were analyzed and processed using ImageJ software to calculate the relative fluorescence density of Nrf2 in the nucleus.

### 2.9. Western Blot

Proteins were extracted from the ischemic penumbra of rat brains and cells via the lysis buffer (Beyotime, P0013), and the supernatant was collected. A BCA kit (Thermo Scientific Company, batch number:23225) was used to measure the protein concentration. Sample buffer (Beyotime, P0013P0015L) was added, and samples were heated to 100°C for 10 min to denature the proteins. After electrophoresis, membrane transfer, and blocking, the membrane was incubated with primary antibodies (NLRP3 [1 : 1000, Cell Signaling Technology, batch number: 13158S], Caspase-1[1 : 1000, Cell Signaling Technology, batch number: 2225S], IL-1*β* [1 : 1000, Abcam, No. 660091.1.g], GSDMD [1 : 1000, Cell Signaling Technology, batch number: 39754S], Nrf2 [1 : 1000, Affinity Biosciences, batch number: AF0639], lamin B1 [1 : 1000, Cell Signaling Technology, batch number: 17416S], and *β*-actin [1 : 8000, Sigma, batch number: A5441) at 4°C overnight. After washing the membrane with TBST five times, membranes were incubated with secondary antibodies (goat anti-rabbit [1 : 10000, Merck Millipore, batch number: ZK-0295G-SA] or goat anti-mouse [1 : 10000, Merck Millipore, batch number: ZK-0293P-HRP]) for 1 h at room temperature. Protein bands were visualized with ECL solution. Quantity One was used for quantitative analysis. The relative integral optical density (IOD) of the target protein was determined with *β*-actin or lamin B1 as the internal reference protein. The ratio of the target protein IOD to the internal reference protein IOD was calculated to represent the relative content of the protein.

### 2.10. Statistical Analysis

SPSS 23.0 software was used for statistical analysis. All of the experimental data are expressed as mean ± standard deviation (SD). One-way ANOVA was used for intergroup comparisons of normally distributed data, and the least significant difference (LSD) method was used for further pairwise comparison of data with homogeneous variance. Unequal variances were tested by the Dunnett T3 test. Nonnormally distributed data were analyzed using the rank-sum test. *P* < 0.05 was considered to indicate a significant difference.

## 3. Results

### 3.1. AST IV Treatment Reduced Cerebral Ischemia-Reperfusion Injury

**Figure 1:**
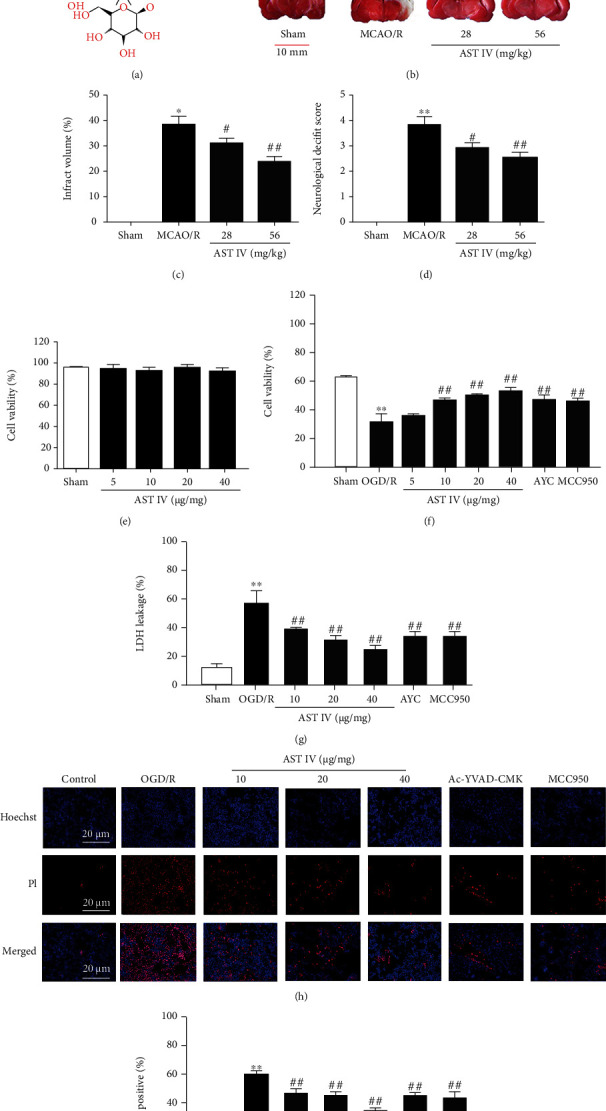
AST IV attenuates CIRI. (a) Chemical structure of AST IV. (b) Brain tissue slices stained with TTC to visualize the ischemic cerebral infarct volume. All rats were randomly assigned to the following groups: the sham group (natural saline, 10 ml/kg), the MCAO/R group (rats given normal saline), and MCAO/R + AST IV groups (AST IV was administered on three successive days at each indicated time, MCAO/R surgery was performed 2 h after the final administration, and AST IV was administered at 24 h just after MCAO/R to achieve reperfusion). (c) Representative quantification of brain infraction volume. Scale bar, 10 mm. Values are mean ± SD, *n* = 10. ^∗∗^*P* < 0.01 vs. the sham group; ^#^*P* < 0.05, ^##^*P* < 0.01 vs. the MCAO/R group. (d) Neurological deficit scores. Values are mean ± SD, *n* = 10. (e) Cell viability was determined with CCK8 assays. Values are mean ± SD, *n* = 4. SH-SY5Y cells were randomly assigned to the following groups: the sham group (complete medium) and the AST IV group (AST IV was administered 24 h at concentrations of 5, 10, 20, and 40 *μ*g/ml). (f) Cell viability was determined with CCK8 assays. Values are mean ± SD, *n* = 4. ^∗∗^*P* < 0.01 vs. the sham group; ^##^*P* < 0.01 vs. the OGD/R group. SH-SY5Y cells were randomly assigned to the following groups: the sham group (complete medium), the OGD/R group (complete medium), OGD/R + AST IV groups (AST IV was given 24 h before OGD, OGD was performed for 2 h, and AST IV was given at 24 h just after OGD to achieve reperfusion), the OGD/R + AC-YVAD-CMK group (30 mM AYC was given 24 h before OGD, OGD was performed for 2 h, and 30 mM AYC was administered at 24 h just after OGD to achieve reperfusion), and the OGD/R + MCC950 group (1 mM MCC950 was given 24 h before OGD, OGD was performed for 2 h, and 1 mM MCC950 was given at 24 h just after OGD to achieve reperfusion). (g) LDH leakage rate was assayed using the LDH Cytotoxicity Detection Kit. Values are mean ± SD, *n* = 4. (h) Hoechst 33342 and PI staining of SH-SY5Y cells. (i) Representative quantification of PI-positive cells. Values are mean ± SD, *n* = 4.

To investigate the neuroprotective effect of AST IV in CIRI, we observed the effects of AST IV on brain injury in MCAO/R Sprague Dawley rats and SH-SY5Y cells treated with OGD/R. In vivo, compared with the sham group, the cerebral infarct volume and neurological deficit scores of rats in the MCAO/R group were significantly higher, while after AST IV (28 mg/kg and 56 mg/kg) intervention, the cerebral infarct volume and neurological deficit scores of rats were markedly decreased (Figures 1(b)–1(d)). In vitro, AST IV at concentrations of 5, 10, 20, and 40 *μ*g/ml had no significant effect on cell viability, indicating that AST IV in this concentration range exerted no cytotoxic effects (Figures 1(e)–1(i)). After OGD/R, cell viability was significantly reduced, and the LDH leakage rate and the percentage of PI-positive cells were increased, while AST IV at 10, 20, and 40 *μ*g/ml remarkably reversed these effects. These findings indicated that AST IV could alleviate CIRI.

In addition, our data indicated that after OGD/R, the Caspase-1 inhibitor AYC and the NLRP3 inhibitor MCC950 substantially increased cell viability and reduced the LDH leakage rate and the percentage of PI-positive cells, demonstrating that inhibition of NLRP3 and Caspase-1 in the NLRP3/Caspase-1/GSDMD pathway could inhibit pyroptosis, reducing cell damage.

### 3.2. AST IV Ameliorated CIRI through Inhibiting the NLRP3/Caspase-1/GSDMD Pathway

**Figure 2:**
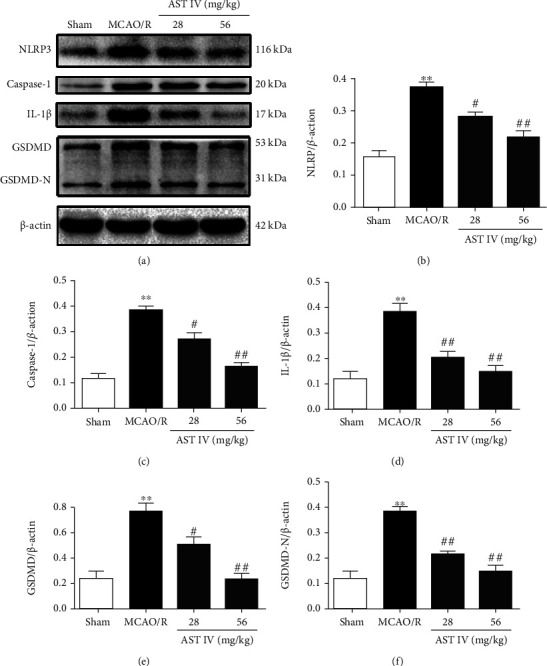
Effect of AST IV on the NLRP3/Caspase-1/GSDMD pathway in MCAO/R rats. (a) Western blot assay showing the expression levels of NLRP3, Caspase-1, IL-1*β*, GSDMD, and GSDMD-N. (b)–(f) Quantitative analysis of NLRP3, Caspase-1, IL-1*β*, GSDMD, and GSDMD-N levels normalized to *β*-actin. Values are mean ± SD, *n* = 5. ^∗∗^*P* < 0.01 vs. the sham group; ^#^*P* < 0.05, ^##^*P* < 0.01 vs. the MCAO/R group.

**Figure 3:**
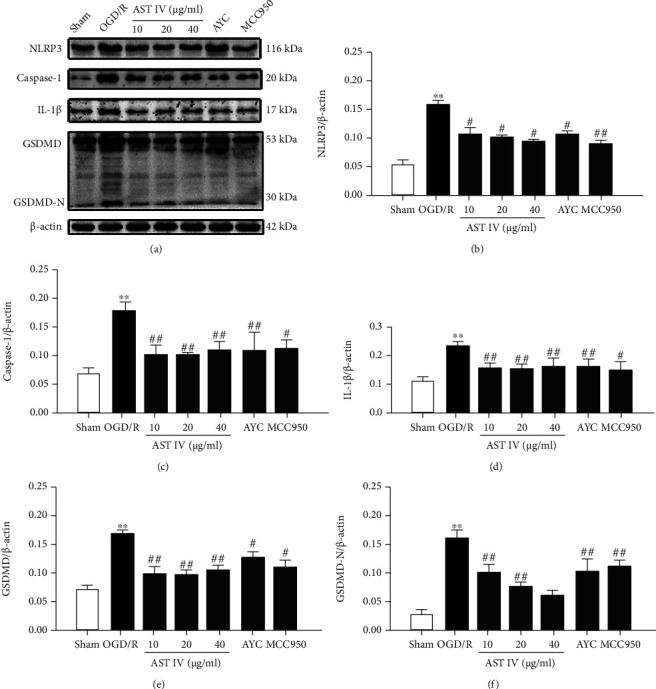
Effect of AST IV on the NLRP3/Caspase-1/GSDMD pathway in OGD/R SH-SY5Y cells. (a) Western blot assay showing the expression levels of NLRP3, Caspase-1, IL-1*β*, GSDMD, and GSDMD-N. (b)–(f) Quantitative analysis of NLRP3, Caspase-1, IL-1*β*, GSDMD, and GSDMD-N levels normalized to *β*-actin. Values are mean ± SD, *n* = 3. ^∗∗^*P* < 0.01 vs. the sham group; ^#^*P* < 0.05, ^##^*P* < 0.01 vs. the OGD/R group.

To determine whether the neuroprotective mechanism of AST IV is related to the inhibition of the NLRP3/Caspase-1/GSDMD pathway, we detected the effects of AST IV on the expression levels of key proteins including NLRP3, Caspase-1, IL-1*β*, GSDMD, and GSDMD-N by Western blot. The levels of NLRP3, Caspase-1, IL-1*β*, GSDMD, and GSDMD-N in the MCAO/R rats were significantly increased. Conversely, AST IV (28 and 56 mg/kg) strongly reduced the above protein levels (Figures 2(a)–2(f)). Similar results were obtained for the OGD/R cell model: NLRP3, Caspase-1, IL-1*β*, GSDMD, and GSDMD-N expression levels were markedly increased after OGD/R treatment, whereas 10, 20, and 40 *μ*g/ml AST IV reversed this effect (Figures 3(a)–3(f)). These results indicated that AST IV reduces NLRP3, Caspase-1, IL-1*β*, GSDMD, and GSDMD-N levels in CIRI and suggested that AST IV inhibits the activation of NLRP3/Caspase-1/GSDMD pathway in CIRI so as to inhibit pyroptosis.

Our in vitro results showed that AYC intervention significantly decreased the levels of Caspase-1, IL-1*β*, GSDMD, and GSDMD-N, while MCC950 intervention diminished the expression levels of NLRP3, Caspase-1, IL-1*β*, GSDMD, and GSDMD-N.

### 3.3. AST IV Facilitated Nrf2 Activation in CIRI

**Figure 4:**
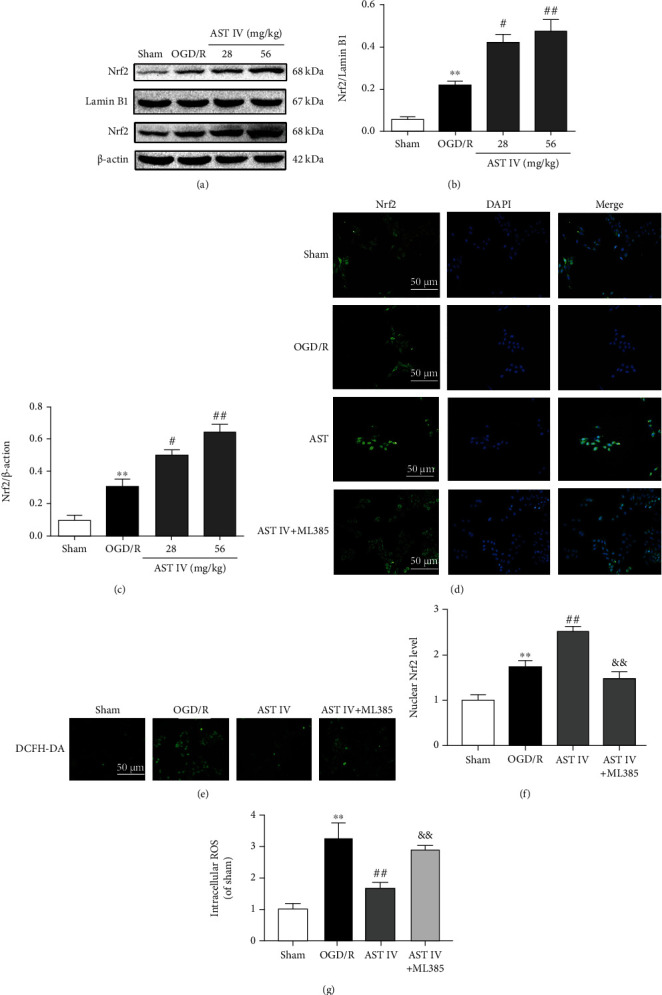
AST IV promotes Nrf2 activation in CIRI. (a) Western blot assay showing the Nrf2 levels in the nuclear and total protein fractions. (b, c) Quantitative analysis of Nrf2 levels in the nuclear, normalized to Lamin B1 and total protein fractions, normalized to *β*-actin. Values are mean ± SD, *n* = 3. ^∗∗^*P* < 0.01 vs. the sham group; ^#^*P* < 0.01, ^##^*P* < 0.01 vs. the MCAO/R group. (d) Immunofluorescence imaging of Nrf2 nuclear colocalization. SH-SY5Y cells were randomly assigned to the following groups: the sham group (complete medium), the OGD/R group (complete medium), the OGD/R + AST IV group (20 *μ*g/ml AST IV was administered 24 h before OGD, OGD was performed for 2 h, and 20 *μ*g/ml AST IV was administered at 24 h just after OGD to achieve reperfusion), and the OGD/R +20 *μ*g/ml AST IV+20 *μ*M ML385 group (20 *μ*g/ml AST IV+20 *μ*M ML385) was administered 24 h before OGD, OGD was performed for 2 h, and 20 *μ*g/ml AST IV+20 *μ*M ML385 was administered at 24 h just after OGD to achieve reperfusion. (e) Intracellular ROS stained via DCFH-DA. (f) Quantitative analysis of Nrf2 nuclear immunofluorescence intensity. Values are mean ± SD, *n* = 3. ^∗∗^*P* < 0.01 vs. the sham group; ^##^*P* < 0.01 vs. the OGD/R group; ^&&^*P* < 0.01 vs. the AST IV group. (g) Representative quantification of cellular ROS contents. Values are mean ± SD, *n* = 3. ^∗∗^*P* < 0.01 vs. the sham group; ^##^*P* < 0.01 vs. the OGD/R group; ^&&^*P* < 0.01 vs. the AST IV group.

Nrf2 is a key factor that negatively regulates the activation of the NLRP3/Caspase-1/GSDMD pathway and protects against CIRI. To explore whether Nrf2 mediates the protective effects of AST IV on CIRI and its inhibitory effects on the activation of NLRP3/Caspase-1/GSDMD pathway, we first observed the effect of AST IV on Nrf2 activation in CIRI rats. Western blot was used to detect the nuclear protein and total protein levels of Nrf2 in brain tissue. Cellular immunofluorescence was used to analyze the nuclear localization of Nrf2. The cellular ROS content was determined using the DCFH-DA kit. Compared with the sham group, the Nrf2 levels in the total protein and nuclear protein fractions of the brain tissue in the MCAO/R group were significantly increased, and intervention with 28 and 56 mg/kg AST IV increased the levels of Nrf2 in the total protein and nuclear protein fractions of the brain tissue in rats (Figures 4(a)–4(c)). Similarly, OGD/R treatment promoted the entry of Nrf2 into the nucleus and increased cellular ROS content in vitro. Nevertheless, AST IV markedly promoted the entry of Nrf2 into the nucleus. Cellular ROS content was significantly decreased in SH-SY5Y cells treated with AST IV. Moreover, the Nrf2-specific inhibitor ML385 could partly block the effect of AST IV (Figures 4(d)–4(g)). These data demonstrated that AST IV facilitates Nrf2 activation in CIRI.

### 3.4. AST IV Prevented against CIRI through NLRP3/Caspase-1/GSDMD Inhibition via Activating Nrf2

**Figure 5:**
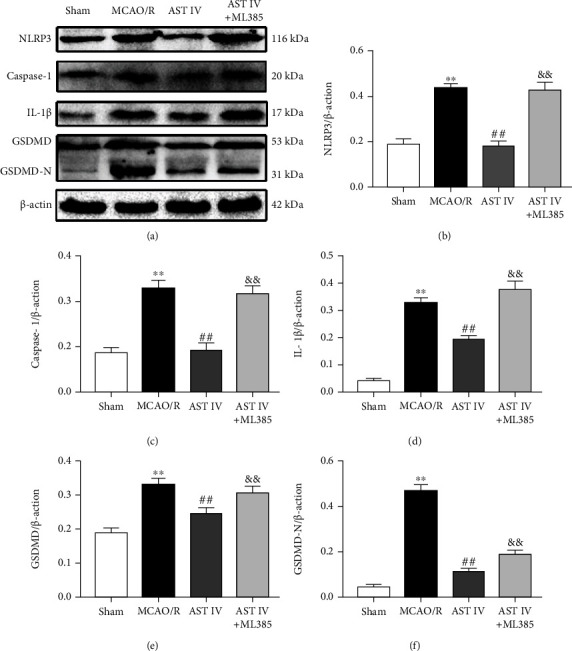
Effect of AST IV on the NLRP3/Caspase-1/GSDMD pathway via Nrf2 in MCAO/R rats. (a) Western blot assay showing the expression levels of NLRP3, Caspase-1, IL-1*β*, GSDMD, and GSDMD-N. (b)–(f) Quantitative analysis of the expression levels of NLRP3, Caspase-1, IL-1*β*, GSDMD, and GSDMD-N normalized to *β*-actin. Values are mean ± SD, *n* = 5. ^∗∗^*P* < 0.01 vs. the Sham group; ^##^*P* < 0.01 vs. the MCAO/R group; ^&&^*P* < 0.01 vs. the AST IV group.

**Figure 6:**
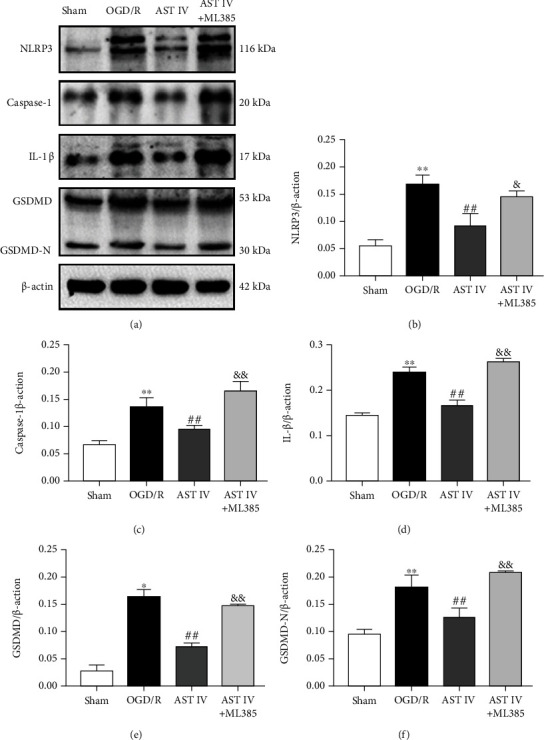
Effect of AST IV on the NLRP3/Caspase-1/GSDMD pathway via Nrf2 in OGD/R SH-SY5Y cells. (a) Western blot assay showing the expression levels of NLRP3, Caspase-1, IL-1*β*, GSDMD, and GSDMD-N. (b)–(f) Quantitative analysis of the expression levels of NLRP3, Caspase-1, IL-1*β*, GSDMD, and GSDMD-N normalized to *β*-actin. Values are mean ± SD, *n* = 3. ^∗^*P* < 0.05. ^∗∗^*P* < 0.01 vs. the sham group; ^#^*P* < 0.05, ^##^*P* < 0.01 vs. the OGD/R group; ^&^*P* < 0.05, ^&&^*P* < 0.01 vs. the AST IV group.

**Figure 7:**
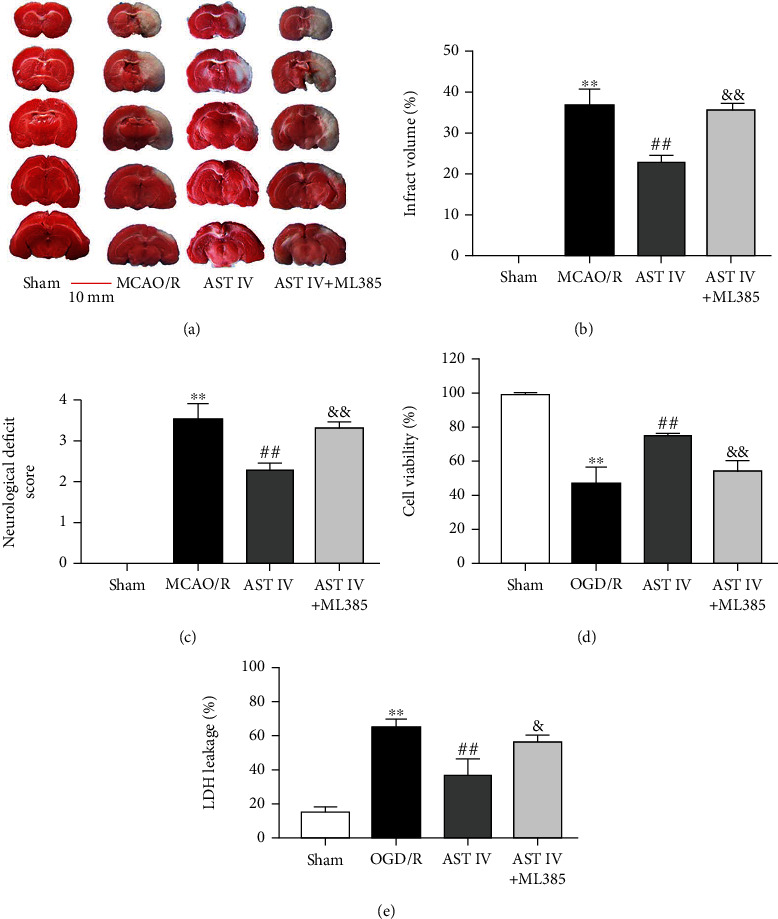
AST IV alleviates CIRI by activating Nrf2. (a) Brain section slices stained with TTC to visualize the ischemic cerebral infarct volume. Scale bar, 10 mm. All rats were randomly assigned to the following groups: the sham group (natural saline, 10 ml/kg), the MCAO/R group (normal saline), and the MCAO/R + AST IV group (AST IV was administered on three successive days at each indicated time, MCAO/R surgery was performed 2 h after the final administration, and AST IV was administered at 24 h just after MCAO/R to achieve reperfusion). (b) Quantitative analysis of brain infarct volume. Values are mean ± SD, *n* = 10. ^∗∗^*P* < 0.01 vs. the sham group; ^##^*P* < 0.01 vs. the MCAO/R group;^&&^*P* < 0.01 vs. the AST IV group. (c) Neurological deficit scores. Values are mean ± SD, *n* = 10. (d) Cell viability was determined with CCK8 assays. Values are mean ± SD, *n* = 4. ^∗∗^*P* < 0.01 vs. the sham group; ^##^*P* < 0.01 vs. the OGD/R group; ^&^*P* < 0.05, ^&&^*P* < 0.01 vs. the AST IV group. (e) LDH leakage rate was assayed using the LDH Cytotoxicity Detection Kit. Values are mean ± SD, *n* = 4.

To examine whether AST IV attenuates CIRI by activating Nrf2 to inhibit the NLRP3/Caspase-1/GSDMD pathway, we conducted Western blot analysis to detect the expression levels of NLRP3, Caspase-1, IL-1*β*, GSDMD, and GSDMD-N after treatment with AST IV or both AST IV and ML385. The protein levels of NLRP3, Caspase-1, IL-1*β*, GSDMD, and GSDMD-N in rats after MCAO/R were significantly increased, while they were markedly decreased after AST IV intervention, which could be blocked by ML385 (Figures 5(a)–5(f)). Likewise, compared with the sham group, the NLRP3, Caspase-1, IL-1*β*, GSDMD, and GSDMD-N protein levels in the OGD/R group were remarkably increased, while AST IV intervention could strongly reduce the above protein levels in vitro, and ML385 could block this effect, indicating that AST IV inhibited the NLRP3/Caspase-1/GSDMD pathway by activating Nrf2 (Figures 6(a)–6(f)). Besides, we also observed the cerebral infarct volume, the neurological deficit score, cell viability, and the LDH leakage rate after treatment with AST IV or both AST IV and ML385. AST IV strongly reduced the cerebral infarct volume and neurological deficit score in rats, improved cell viability, and decreased the LDH leakage rate. After combined treatment with ML385 and AST IV, the effect of AST IV was reversed. These results suggested that AST IV protects against CIRI through inhibiting the NLRP3/Caspase-1/GSDMD pathway via activating Nrf2 (Figure 7).

## 4. Discussion

We demonstrated that AST IV has the ability to reduce the cerebral infarct volume and the neurological deficit score in MCAO/R rats, increase the cell viability of SH-SY5Y cells induced by OGD/R, and decrease the LDH leakage rate and the proportion of PI-positive cells, indicating that AST IV is capable of reducing CIRI in vitro and in vivo. Previous studies revealed that AST IV plays a protective role in OGD/R-induced cells and MCAO/R-induced rats, relieving CIRI [23–26], implying that AST IV is able to protect against CIRI.

The pathological mechanisms underlying CIRI are complicated, and they are related to a variety of cell death modes. It has been shown that the NLRP3/Caspase-1/GSDMD pathway and pyroptosis are activated in CIRI, aggravating injury [5]. Additionally, pyroptosis mediated by the NLRP3 inflammasome has been shown to participate in and exacerbate CIRI. The present study revealed that the expression of proteins in the NLRP3/Caspase-1/GSDMD pathway was markedly increased in an MCAO/R-induced rat model and an OGD/R-induced cell culture model. Furthermore, our in vitro results revealed that the LDH leakage rate and the proportion of PI-positive cells increased in OGD/R-induced cells, whereas the proteins NLRP3 and Caspase-1 in the inhibitory pathway reduced the levels of related proteins, increasing cell viability and reducing the LDH leakage rate and the proportion of PI-positive cells, indicating that ischemia-reperfusion activates the NLRP3/Caspase-1/GSDMD pathway and pyroptosis, causing CIRI. Previous studies revealed that inhibition of the NLRP3/Caspase-1/GSDMD pathway inhibited NLRP3 inflammasome-mediated pyroptosis and alleviated CIRI. We provided significant evidence that inhibition of NLRP3 and Caspase-1 reduced the levels of key proteins in the pathway, inhibiting the activation of the NLRP3/Caspase-1/GSDMD pathway and preventing pyroptosis. Specifically, AST IV played a regulatory role in reducing the levels of key proteins in the NLRP3/Caspase-1/GSDMD pathway, the LDH leakage rate, and the proportion of PI-positive cells. This suggests that AST IV could prevent the activation of the NLRP3/Caspase-1/GSDMD pathway and pyroptosis to alleviate CIRI. This study concerns neurons after CIRI. Presently, microglia activation is also an important factor that causes pyroptosis and leads to neurological damage after CIRI. Therefore, in order to further study the cascade mechanism of the CIRI inflammatory response, how microglial cells induce pyroptosis to accelerate the inflammatory response and thus aggravate brain injury needs further consideration and discussion [35, 36].

Nrf2 plays an antioxidative stress role primarily by upregulating the expression of various antioxidant enzymes and phase II detoxification enzymes in the body, inhibiting the antiapoptosis of the NOX4/ROS/NF-*κ*B pathway, regulating the activity of inflammatory cytokines and anti-inflammation, and activating the Nrf2-VEGF pathway to promote angiogenesis in CIRI [37, 38]. Studies have shown that Nrf2 is capable of negatively regulating the activation of the NLRP3/Caspase-1/GSDMD pathway and inhibiting pyroptosis [16–18], and that AST IV activates Nrf2 to alleviate CIRI [39]. To further explore whether Nrf2 mediates the regulatory effects of AST IV on the NLRP3/Caspase-1/GSDMD pathway, we observed the effects of AST IV on Nrf2 activation. Our data showed that AST IV (i) has the ability to increase the total and nuclear protein levels of Nrf2 in MCAO/R rats and (ii) stimulates Nrf2 translocation into the nucleus in SH-SY5Y cells induced by OGD/R, reducing the intracellular ROS levels, confirming that AST IV takes part in promoting Nrf2 activation upon cerebral ischemia-reperfusion.

Also, our results revealed that the regulatory effects of AST IV on ROS, key proteins in the NLRP3/Caspase-1/GSDMD pathway, and CIRI could be blocked by the Nrf2 inhibitor ML385, indicating that Nrf2 is involved in the negative regulatory effects of AST IV on NLRP3 inflammasome-mediated pyroptosis to alleviate CIRI. Nevertheless, the specific mechanism by which AST IV activates Nrf2 remains to be determined.

## 5. Conclusion

Our study revealed that AST IV alleviates CIRI through inhibiting NLRP3 inflammasome-mediated pyroptosis via activating Nrf2.

## Data Availability

The datasets generated for this study are available upon request to the corresponding author.
